# TLR4 Endogenous Ligand S100A8/A9 Levels in Adult-Onset Still’s Disease and Their Association with Disease Activity and Clinical Manifestations

**DOI:** 10.3390/ijms17081342

**Published:** 2016-08-16

**Authors:** Hyoun-Ah Kim, Jae Ho Han, Woo-Jung Kim, Hyun Jin Noh, Jeong-Mi An, Hyunee Yim, Ju-Yang Jung, You-Sun Kim, Chang-Hee Suh

**Affiliations:** 1 Department of Rheumatology, Ajou University School of Medicine, 164 Worldcup-ro, Yeongtong-gu, Suwon 443-380, Korea; nakhada@naver.com (H.-A.K.); godofpeace@nate.com (J.-M.A.); serinne20@hanmail.net (J.-Y.J.); 2 Department of Pathology, Ajou University School of Medicine, 164 Worldcup-ro, Yeongtong-gu, Suwon 443-380, Korea; hanpathol@naver.com (J.H.H.); Huuneeyim@naver.com (H.Y.); 3 Department of Biochemistry and Department of Biomedical Sciences, Ajou University School of Medicine, 164 Worldcup-ro, Yeongtong-gu, Suwon 443-380, Korea; kwj881010@hanmail.net (W.-J.K.); simuner@naver.com (H.J.N.)

**Keywords:** adult-onset Still’s disease, S100A8/A9, interleukin-1β, biomarker, disease activity

## Abstract

S100A8/A9 has been suggested as a marker of disease activity in patients with adult-onset Still’s disease (AOSD). We evaluated the clinical significance of S100A8/A9 as a biomarker and its pathogenic role in AOSD. Blood samples were collected prospectively from 20 AOSD patients and 20 healthy controls (HCs). Furthermore, skin and lymph node biopsy specimens of AOSD patients were investigated for S100A8/A9 expression levels via immunohistochemistry. Peripheral blood mononuclear cells (PBMCs) of active AOSD patients and HCs were investigated for S100A8/A9 cell signals. S100A8/A9, interleukin-1β (IL-1β), and tumor necrosis factor-α (TNF-α) levels in active AOSD patients were higher than those of HCs. S100A8/A9 levels correlated positively with IL-1β, TNF-α and C-reactive protein. The inflammatory cells expressing S100A8/A9 were graded from one to three in skin and lymph node biopsies of AOSD patients. The grading for S100A8/A9 was more intense in the skin lesions with karyorrhexis, mucin deposition, and neutrophil infiltration. Like lipopolysaccharide (LPS), S100A8/A9 induced phosphorylation of p38 and c-Jun amino-terminal kinase (JNK) in PBMCs, suggesting that S100A8/A9 activates Toll-like receptor 4 signaling pathways. These findings suggest that S100A8/A9 may be involved in the inflammatory response with induction of proinflammatory cytokines and may serve as a clinicopathological marker for disease activity in AOSD.

## 1. Introduction

Adult-onset Still’s disease (AOSD) is an uncommon systemic inflammatory disease of unknown cause. Its clinical manifestations are characterized by a high repetitive spiking fever, an evanescent skin rash, myalgia, lymphadenopathy, and hepatosplenomegaly [1]. Although the initial manifestations of all patients are similar, with systemic symptoms, the clinical course of the patients proceeds according to three distinct patterns: monocyclic, polycyclic, and chronic articular patterns [1,2]. Although the pathogenesis of AOSD remains unclear, multiple factors including genetic background, viral infections, and aberrant immune response have been suggested to be involved in the development of the disease [3,4]. Genetically predisposed hosts seem to develop auto-inflammatory conditions triggered by macrophage activation and Th1 cytokines, such as tumor necrosis factor-α (TNF-α), interleukin-1 (IL-1), IL-6, IL-8, and IL-18 [5].

Proinflammatory cytokines, such as IL-1β and TNF-α are amplified by endogenous factors, like S100 proteins. These proteins induce inflammatory responses through the recruitment of inflammatory cells and are suggested to be one of the “damage-associated molecular patterns” [6,7]. S100A8 and S100A9, which belong to the S100 family, are calcium-binding proteins and form heterodimers that are the biologically relevant form [8]. S100A8/A9 is produced by infiltrating neutrophils and monocytes but not by quiescent resident macrophages or lymphocytes under inflammatory status [9,10]. S100A8/A9 has been shown to be an endogenous ligand of Toll-like receptor-4 (TLR-4), to be associated with human sepsis and endotoxemia, and to play an important role in innate immunity [10,11,12].

S100A8/A9 has been detected at high levels in the serum and body fluids of patients with several inflammatory disorders [11,13,14,15,16]. Some studies showed that systemic juvenile idiopathic arthritis (systemic JIA) is associated with high concentrations of S100A8/A9 [17,18,19]. Furthermore, we showed previously elevated serum S100A8/A9 levels in AOSD patients, compared with healthy controls (HCs), and their correlation with several disease activity markers in AOSD [20].

In this study, we have investigated the correlation between S100A8/A9 and proinflammatory cytokines in active AOSD patients recruited prospectively. Furthermore, to evaluate the in vivo involvement of S100A8/A9 in AOSD, we immunohistochemically stained biopsy specimens obtained from the skin lesions of 26 patients and lymph nodes of eight patients with active untreated AOSD for S100A8/A9. We also investigated the association between IL-1β and S100A8/A9 with peripheral blood mononuclear cells (PBMCs) of active AOSD patients and HCs and a monocyte cell line.

## 2. Results

### 2.1. Clinical Characteristics of the 20 Adult-Onset Still’s Disease (AOSD) Patients, 20 Rheumatoid Arthritis (RA) Patietns, and 20 Healthy Controls (HCs)

The mean age of the AOSD patients was 38 ± 13.7 years, and 85% were female. There were no differences in age or gender among groups (Table 1). The main symptoms in AOSD patients were fever (100%), skin rash (75%), sore throat (55%), arthritis (52.8%), and lymphadenopathy (40%).

### 2.2. Serum S100A8/A9 Levels in AOSD, RA Patients, and HCs

**Figure 1:**
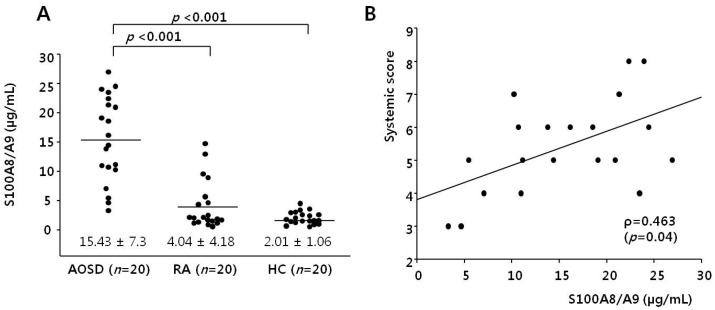
(**A**) Levels of S100A8/A9 in 20 active adult-onset Still’s disease (AOSD) patients, 20 rheumatoid arthritis (RA) patients, and 20 healthy controls (HCs). Data are expressed as the means ± standard deviation (SD). A Mann-Whitney *U*-test was used to perform the statistical analysis; (**B**) the levels of S100A8/A9 correlated with the systemic scores in AOSD patients (*r* = 0.463, *p* = 0.04).

Figure 1A shows S100A8/A9 levels in AOSD patients, RA patients, and HCs. The levels of S100A8/A9 in AOSD patients (15.43 ± 7.3 µg/mL) were higher than those of RA patients and HCs (4.04 ± 4.18 µg/mL, *p* < 0.001; 2.01 ± 1.06 µg/mL, *p* < 0.001, respectively).

We evaluated the correlation between systemic score and serum S100A8/A9 in AOSD patients. Serum S100A8/A9 levels correlated with systemic score (*r* = 0.463, *p* = 0.04; Figure 1B).

### 2.3. Serum Interleukin-1β (IL-1β), and Tumor Necrosis Factor-α (TNF-α) Levels in AOSD Patients and HCs

**Figure 2:**
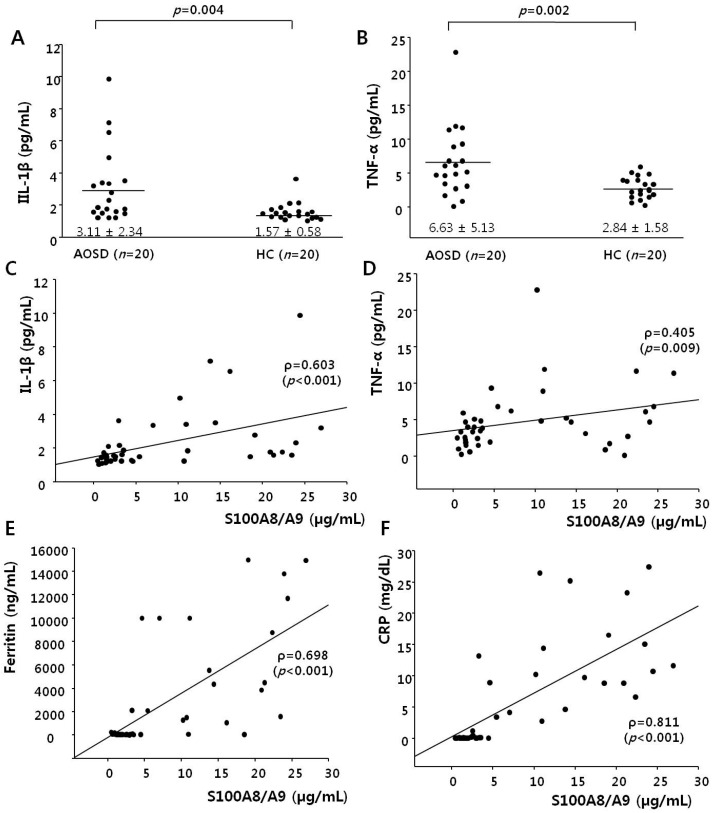
Levels of inteleukin-1β (IL-1β) (**A**) and tumor necrosis factor-α (TNF- α) (**B**) in 20 patients with active adult-onset Still’s disease (AOSD) and 20 healthy controls (HCs). Data are shown as the means ± SD. A Mann-Whitney *U*-test was performed for the statistical analysis. Serum S100A8/A9 levels correlated positively with IL-1β (*r* = 0.603, *p* < 0.001) (**C**), TNF-α (*r* = 0.405, *p* = 0.009) (**D**), ferritin (*r* = 0.698, *p* < 0.001) (**E**), and C-reactive protein (CRP) (*r* = 0.811, *p* < 0.001) (**F**). These data were assessed using Spearman’s correlation.

Figure 2 shows IL-1β and TNF-α levels in AOSD patients and HCs. Serum IL-1β levels of AOSD patients (3.11 ± 2.34 pg/mL) were higher than those of HCs (1.57 ± 0.58, *p* = 0.004), and the TNF-α levels of AOSD patients (6.63 ± 5.13 pg/mL) were also higher than those of HCs (2.84 ± 1.58 pg/dL, *p* = 0.002). We sought to determine whether the levels of IL-1β and TNF-α were associated with the level of S100A8/A9. S100A8/A9 levels correlated positively with IL-1β and TNF-α (*r* = 0.603, *p* < 0.001; *r* = 0.405, *p* = 0.009, respectively). In addition, they correlated positively with ferritin and CRP (*r* = 0.698, *p* < 0.001; *r* = 0.811, *p* < 0.001, respectively).

### 2.4. IL-1β Secretion after Treatment of S100A9 in PBMCs from Active AOSD Patients and HCs

**Figure 3:**
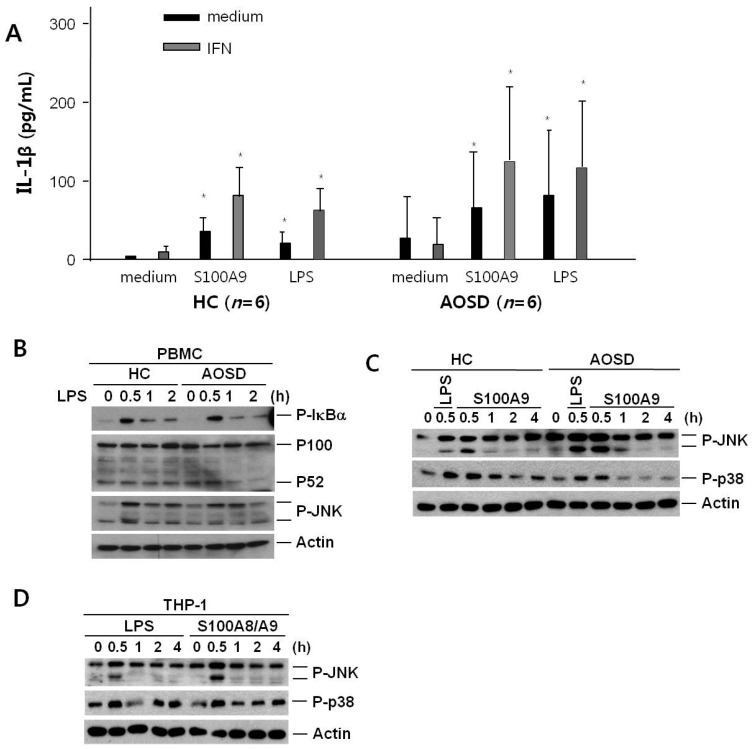
(**A**) Interleukin-1β (IL-1β) secretion after treatment with S100A9 in peripheral blood monocyte cells (PBMCs) of healthy controls (HCs) and patients with active adult-onset Still’s disease (AOSD) patients. PBMCs (1 × 10^6^/mL) were incubated for 24 h with 5 µg/mL S100A9 or 10 ng/mL lipopolysaccharide (LPS) or left untreated as controls (medium). Concentrations of IL-1β in supernatants were evaluated by enzyme-linked immunosorbent assay. Data are shown from six independent experiments. Values are the means and SD. * *p* ≤ 0.05, vs. controls; (**B**) Activation of c-Jun amino-terminal kinase (JNK) and p38 after treatment with S100A9 in PBMCs from HCs and patients with active AOSD. PBMCs were treated with LPS or S100A9 for the indicated time. For immunoblot analysis, total cellular proteins were extracted. p100, p52, phosphorylated IκBα, and JNK in PBMC from HC and AOSD treated with LPS; (**C**) Phosphorylated JNK and p38 in PBMCs from HCs and AOSD patients treated with LPS and S100A9; (**D**) Activation of JNK and p38 in a human monocyte cell line after treatment with S100A8/A9. THP-1 cells were treated with S100A8/A9 or LPS for the indicated time. For immunoblot analysis, total cellular proteins were extracted. Phosphorylated JNK and p38 in THP-1 cells treated with S100A8/9.

We thought that monocytes could be major cells for the inflammatory condition in AOSD. Thus, we stimulated PBMCs from six AOSD patients and six HCs in vitro with S100A9, and evaluated IL-1β levels. Stimulation of PBMCs from AOSD patients and HCs in vitro revealed that S100A9 was an inducer of IL-1β secretion, with levels comparable to those observed with lipopolysaccharide (LPS) (Figure 3A). Priming of PBMCs with interferon gamma (IFN-γ) augmented the effects of both S100A9 and LPS in PBMCs from six AOSD patients and six HCs. However, the secreted levels of IL-1β from the PBMCs of the AOSD patients were not elevated significantly compared with PBMCs from the HCs in medium, S100A9, or LPS.

### 2.5. Expression of c-JUN Amino-Terminal Kinase (JNK) and p38 after Treatment with S100A9 in PBMCs from Active AOSD Patients and HCs and in THP-1 Cells

We sought to determine the mechanism by which the endogenous TLR4 ligand S100A8/A9 induced IL-1β in monocytes. First, we evaluated several transcription factors, such as p100, phosphorylated IκBα, and JNK, in monocytes/macrophages treated with LPS, an exogenous TLR4 ligand. Immunoblot analysis was performed with antibodies specific to p100, p52, phosphorylated IκBα, and JNK in PBMCs from AOSD patients and HCs treated with LPS for 2 h (Figure 3B). IκBα and JNK were significantly more phosphorylated at 0.5 h in PBMCs from AOSD patients and HCs with LPS. In addition, phosphorylated IκBα and JNK were similar to PBMCs of AOSD patients and HCs treated with LPS. However, p100 and p52 were not different for 2 h, and these data were similar between AOSD patients and HCs. We next evaluated phosphorylated JNK and p38 from PBMCs of AOSD patients and HCs with S100A9 compared with LPS. Immunoblot analysis was performed with antibodies specific to phosphorylated JNK and p38 in PBMCs from HCs and AOSD patients treated with S100A9 or LPS for 4 h (Figure 3C). JNK and p38 were significantly more phosphorylated at 0.5 h in PBMCs from HCs and AOSD patients treated with S100A9, similar to those treated with LPS.

We evaluated activation of the transcription factors with monocytes/macrophages. We assayed with THP-1 cells treated with S100A8/A9 and LPS, and confirmed that JNK and p38 were significantly phosphorylated with S100A8/A9 at 0.5 h, which was similar to those treated with LPS (Figure 3D).

### 2.6. Histopathological and Immunohistochemical Characteristics of Skin and Lymph Nodes in AOSD

Immunohistochemical analyses of skin and lymph nodes were performed for S100A8/A9 to evaluate the organ involvement of this endogenous TLR4 ligand in AOSD. Most skin lesions showed mild histiocytic or lymphocytic infiltration in the upper dermis. More than half the cases had mucin deposition in interstitium. Nuclear debris was observed in the dermis in 14 (53.8%) cases. Eight lymph node biopsy revealed only paracortical hyperplasia (*n* = 5) and a mixed pattern (*n* = 3) with paracortical and diffuse (*n* = 2) and paracortical, follicular and diffuse (*n* = 1). Vascular proliferation was shown from moderate to severe in all lymph nodes, and immunoblast proliferation was observed moderate-to-severe in five (62.5%) lymph nodes.

**Figure 4:**
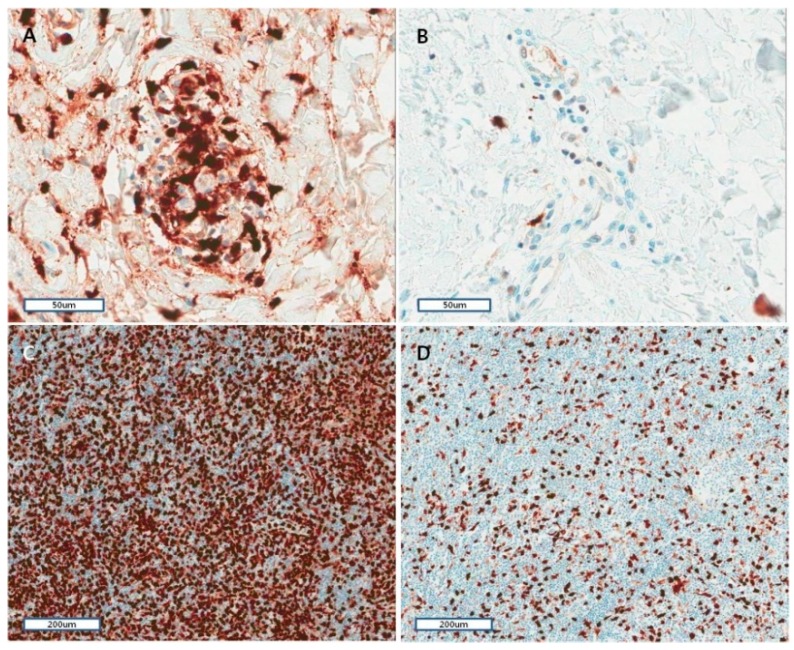
S100A8/A9 expression levels of inflammatory cells in skin and lymph node biopsy of patients with active adult-onset Still’s disease (original magnification, 200× (**A**,**B**), 100× (**C**,**D**)). S100A8/A9 was recorded as the number of positive inflammatory cells divided by the number of total inflammatory cells, then expressed as a graded scale from 1 to 3: 1, 1%–33%, 2, 34%–66%, or 3, 67%–100%. The S100A8/A9 staining positive cells were graded from 1 to 3. Representative examples of frequent expression (grade 3, **A**) and rare expression (grade 1, **B**) are shown in the skin. Representative examples of frequent expression (grade 3, **C**) and rare expression (grade 1, **D**) are shown in the lymph node.

Lymphoid cells of the paracortical zone in a tonsil were stained for control for S100A8 and S100A9 immunohistochemical explorations. The antibodies showed a diffuse or granular cytoplasmic pattern. The patterns of inflammatory cells staining for skin biopsies were similar to those of the lymphoid cells in a tonsil (Figure 4A,B). The S100A8/A9 staining positive cells were graded from one to three. The grading of S100A8/A9 staining positive cells was higher when there was karyrrhexis, mucin deposition, and neutrophil infiltration (Table 2). Furthermore, the correlation between inflammatory cell grading of Cluster of Differentiation (CD)68 and that of S100A8/A9 was significant (*p* < 0.001), but was not significant between CD4 or CD8 and that of S100A8/A9.

The S100A8/A9 staining positive cells were graded from one to three in lymph node biopsies (Figure 4C,D). The grading and strength of S100A8/A9 staining positive cells was marginally more intense for severe vascular proliferation (*p* = 0.05).

## 3. Discussion

Serum S100A8/A9 levels of patients with active untreated AOSD were higher than those of RA patients and HCs, and showed correlations with IL-1β and TNF-α. In addition, S100A8/A9 was immunohistochemically stained in lesional skin and lymph nodes from AOSD patients. Moreover, it was found that S100A9 induced IL-1β expression in monocytes from AOSD patients and HCs. Furthermore, S100A9 and S100A8/A9 were shown to induce signal transduction pathways, including p38 and JNK in PBMCs of AOSD patients, HCs, and THP-1 cells.

S100A8/A9 is known as an intracellular differentiation marker for phagocytes but also as an extracellular protein complex involved in inflammatory response, i.e., one of the damage-associated molecular patterns molecules (DAMPs) [21]. S100A8/A9 has shown to be a biomarker of several inflammatory diseases including RA, systemic JIA, inflammatory bowel disease, and AOSD [19,20,22,23,24]. One study showed that serum S100A8/A9 levels were higher in patients with active systemic JIA, compared with those with systemic infections and HCs [18]. In addition, S100A8/A9 discriminated systemic JIA from systemic infections with high specificity, in contrast to CRP levels. A recent study confirmed that serum S100A8/A9 levels correlated well with response to treatment, and suggested that it might be an excellent biomarker for monitoring the treatment in systemic JIA [17]. In this study, we recruited active untreated AOSD patients, RA patients, and HCs prospectively, and evaluated S100A8/A9 with proinflammatory cytokines, such as IL-1β and TNF-α. Serum S100A8/A9 levels of patients with AOSD were significantly higher than those of patients with RA and HCs. Additionally, serum S100A8/A9 levels showed strong positive correlations with several inflammatory markers, such as CRP, ferritin, IL-1β, and TNF-α. We confirmed again that S100A8/A9 could provide reliable clinical information for monitoring disease activity of AOSD. 

In addition to the clinical implications of S100A8/A9 in AOSD, this data showed the important role of innate immune processes involving S100A8/A9 in the pathogenesis of AOSD. We confirmed that S100A9 was a strong inducer of IL-1β expression in monocytes of AOSD patients and HCs. In addition, we showed that S100A9 induced the phosphorylation of p38 and JNK in PBMCs of AOSD patients and HCs. These results were consistent with previous reports [25,26]. S100A8/A9 is known as an endogenous TLR4 ligand, similar to LPS [10]. S100A8/A9 is also an inducer for a thrombogenic response in human microvascular endothelial cells by decreasing the expression of cell junction proteins and molecules and by increasing the transcription of proinflammatory cytokines or adhesion molecules [27]. The elevation of S100A8/9, an internal TLR4 ligand, in AOSD may suggest a similarity in inflammatory responses and disease manifestations between AOSD and septic conditions. However, IL-1β expression in phagocytes of AOSD patients was not significantly different from that in phagocytes from HCs, and the findings were consistent in the expression of transcription factors between AOSD patients and HCs.

We analyzed the expression of S100A8/A9 during the initial phase of AOSD using skin and lymph node biopsy specimens obtained before treatment. S100A8/A9 was expressed in skin and lymph nodes of patients with AOSD. Such staining of S100A8/A9 was correlated significantly with the CD68-stained inflammatory cells in the skin of AOSD. Furthermore, enhanced S100A8/A9 staining was evident in neutrophil infiltration and inflammatory skin lesions with mucin deposition and karyorrhexis. Although we did not compare inflammatory skin or lymph node lesions from AOSD with those from HCs, S100A8 and S100A9 are known to be expressed with low levels in normal epidermis [21]. S100A8/A9 is highly expressed in the skin lesions of several inflammatory skin diseases including psoriasis, systemic lupus erythematosus, and lichen planus [28,29]. S100A8/A9 could be a part of a positive feedback mechanism in the initiation and amplification of inflammatory skin diseases through inducing proinflammatory cytokine production as well as proliferation of keratinocytes [30]. Furthermore, in psoriasis, epidermal S100A8/A9 overexpression seems to be related to increase serum S100A8/A9 levels, which correlate with disease activity [31]. Epidermal overexpression of S100A8/A9 was observed in typical skin rashes of systemic JIA, and correlated with elevated circulating S100A8/A9 levels [19,32]. In this study, we showed that S100A8/A9 was expressed in skin and lymph nodes affected by AOSD, and correlated with neutrophil infiltration and CD68-stained inflammatory cells. These results could suggest that the infiltrating neutrophils and CD68-positive inflammatory cells are major sources of S100A8/A9-related inflammation. Furthermore, staining of S100A8/A9 was related to skin lesions with mucin deposition and karyorrhexis. These results suggest that S100A8/A9 plays a key role for the skin inflammation in AOSD. However, we could not demonstrate a correlation between S100A8/A9 expression in skin and serum S100A8/A9 levels, because we did not have serum samples from the AOSD patients who underwent biopsies.

## 4. Experimental Sections

### 4.1. Subjects

In total, 20 AOSD patients, 20 rheumatoid arthritis (RA) patients as a disease control, and 20 HCs were prospectively included in this study. AOSD patients satisfied the Yamaguchi’s criteria after the exclusion of viral or bacterial infections, hematologic diseases, and autoimmune disorders [33]. RA patients were diagnosed according to American College of Rheumatology 1987 revised criteria for the classification of RA [34]. Blood samples were collected from patients and controls. All serum samples were stored at −70 °C immediately after collection. This study was approved by the Institutional Review Board of our hospital (AJIRB-MED-SMP-11-094). Informed consent was acquired from all enrolled subjects.

Information of the medical history, clinical manifestations, and physical examinations was obtained when blood sampling was done. Each patient also underwent laboratory tests including complete blood count, erythrocyte sedimentation rate (ESR), C-reactive protein (CRP), ferritin (normal = 13–150 ng/mL for women and 30–400 ng/mL for men), liver function tests, urinalysis, rheumatoid factor, and anti-nuclear antibody. AOSD disease activity was evaluated with a systemic score using the method suggested by Pouchot et al. [35], which adds one point for the following manifestations: fever, rash, sore throat, myalgia, abdominal pain, pneumonia, pleuritis, pericarditis, leukocytosis (≥15,000/mm^3^), hepatomegaly or abnormal liver function tests, splenomegaly, and lymphadenopathy.

### 4.2. S100A8/A9, IL-1β, and TNF-α Assays

Serum S100A8/A9 concentrations were measured with commercial enzyme-linked immunosorbent assay (ELISA) kits (Buhlmann Laboratories, Schonenbuch, Switzerland) according to the manufacturer’s protocol. Serum concentrations of IL-1β and TNF-α were measured using commercial ELISA kits (R&D Systems, Minneapolis, MN, USA) according to the manufacturer’s protocol.

### 4.3. PBMC Preparation and Stimulation of Six Active AOSD Patients and Six HCs

PBMCs were isolated from human buffy coats from six patients with active AOSD and six HCs and cultured. PBMCs were incubated for 24 h with S100A9 (5 µg/mL), S100A8/A9 (5 µg/mL), or LPS (10 ng/mL; Sigma, Deisenhofen, Germany) and IL-1β concentrations in supernatants were measured by ELISA (R&D Systems). The interassay variability of IL-1β baseline production between different PBMC culture is the reason that the results are reported as a percentage of the control. In parallel, PBMCs were primed for 16 h incubated with interferon-γ (500 IU/mL; Bender MedSystems, Vienna, Austria) prior to stimulation with LPS, S100A8/A9, or S100A9.

### 4.4. Cell Culture

THP-1 cells were cultured in RPMI 1640 medium supplemented with 10% FBS, 2 mM glutamine, 10 mM HEPES, 1 mM sodium pyruvate, 0.05 mM 2-mercaptoethanol, and 100 U/mL penicillin and streptomycin. No further authentication of the cell line was performed.

### 4.5. Immunobot

With treatment, cell lines or PBMCs were lysed in 20 mM Tris (pH 7.0), 250 mM NaCl, 3 mM EDTA, 3 mM EGTA, 2 mM DTT, 0.5% NP-40, 0.5 mM PMSF, 20 mM β-glycerol phosphate, 1 mM sodium vanadate and 1 µg/mL leupeptin. Cell lysates were loaded on 10% or 12% SDS-PAGE gels. After transfer and blotting, the target proteins were visualized by enhanced chemiluminescence (Pierce, Rockford, IL, USA) and analyzed.

### 4.6. Histopathology of Skin Biopsy and Lymph Node

Skin biopsies of 26 patients with active AOSD and eight lymph node biopsies were obtained. We investigated the hematoxylin and eosin-stained sections, as previously described [36]. The slides were examined by two pathologists (JHH and HY) independently with the following skin histological parameters of epidermal change, and presence of karyorrhexis, vasculitis, and interstitial mucin, and degree of inflammatory cell infiltration. Lymph node histological parameters were evaluated with patterns of reaction (follicular, paracortical, or diffuse hyperplasia), kinds of infiltrating inflammatory cells, immunoblast proliferation, and vascular proliferation.

### 4.7. Immunohistochemistry and Evaluation

Immunohistochemistry was performed with several primary antibodies on 4-µm representative tissue sections of formalin-fixed, paraffin wax-embedded tissue with the Benchmark XT automated immunohistochemistry stainer (Ventana Medical Systems Inc., Tucson, AZ, USA). The primary antibodies were used with CD4, 1:10 and CD8, 1:50 (Thermo Fisher Scientific, Fremont, CA, USA), CD68, 1:200 (Novocastra Laboratories Ltd., Newcastle, UK), S100A8, 1:300 and S100A9, 1:400 dilution (Abcam Systems, Cambridge, UK). Detection was done using the Ventana Optiview DAB Kit (Ventana Medical Systems). Each marker for immunohistochemical staining was recorded as the number of positive inflammatory cells divided by the number of total inflammatory cells, and expressed as grade 1 to grade 3: 1, 1%–33%, 2, 34%–66%, or 3, 67%–100% (CD4, CD8, CD68, and S100A8/A9).

### 4.8. Statistical Analysis

Statistical analyses were performed using the SPSS for Windows software (ver.12.0; SPSS, Chicago, IL, USA). A *p*-value < 0.05 was regarded as indicating statistical significance. The data are shown as means ± standard deviation (SD) or median and interquartile range, as appropriate. Differences in S100A8/A9 and cytokine levels were determined using the Mann-Whitney *U*-test. The correlations between their levels and disease activity markers were assessed with Spearman’s correlation test.

## 5. Conclusions

We found higher levels of S100A8/A9 in the serum and upon immunohistochemical staining of pathological skin tissue and lymph node from patients with active untreated AOSD. Furthermore, S100A8/A9 correlated with several inflammatory markers and disease activity markers, and this DAMP induced IL-1β production. These findings support the important role of S100A8/A9 in the pathogenesis of AOSD, and may also suggest novel diagnostic or therapeutic strategies.

## Figures and Tables

**Table 1:** Clinical characteristics of patients.

Clinical Manifestations and Laboratory Findings	AOSD (*n* = 20)	RA (*n* = 20)	HC (*n* = 20)
Age (year)	38 ± 13.7	41.8 ± 15.2	39.3 ± 8.3
Gender (F/M)	17/3	18/2	19/1
Fever	20 (100)		
Sore throat	11 (55)		
Skin rash	15 (75)		
Lymphadenopathy	8 (40)		
Splenomegaly	6 (30)		
Hepatomegaly	4 (20)		
Pericarditis	3 (15)		
Pleuritis	2 (10)		
Arthritis	19 (52.8)	40 (100)	
Hemoglobin, g/dL	11.1 ± 1.8		
Leukocyte, /µL	15,554 ± 4553		
Platelet, ×10^3^/µL	324.7 ± 124.4		
Ferritin, ng/mL	6100.4 ± 5158.3	72.7 ± 80.1	54.2 ± 58.4
ESR, mm/h	65.5 ± 22.9	30 ± 33.4	
CRP, mg/dL	10.6 ± 6.8	0.95 ± 1.7	0.09 ± 0.21
AST, U/L	96 ± 125.1		
ALT, U/L	88.7 ± 105		
ANA positivity	3 (15)	4 (20)	
RF positivity	5 (25)	12 (60)	
Systemic score	5.4 ± 1.43		
DAS-28	3.8 ± 1.22	4.05 ± 1.6	

AOSD, adult-onset Still’s disease; RA, rheumatoid arthritis; HC, healthy control; F, female; M, male; ESR, erythrocyte sedimentation rate; CRP, C-reactive protein; AST, aspartate transaminase; ALT, alanine transaminase; ANA, antinuclear antibody; RF, rheumatoid factor; DAS-28, disease activity score including 28 joints. All values presented as means ± SD or number (%).

**Table 2:** The inflammatory cell staining for S100A8/A9 of skin in adult-onset Still’s disease.

Pathologic Finding and S100A8/A9 Staining Grade	Grade 1	Grade 2	Grade 3	*p*-Value
Vacuole				
(+), *n* = 6	2	2	2	0.292
(−), *n* = 20	4	4	12	
Karyorrhexis				
(+), *n* = 14	1	3	10	0.028
(−), *n* = 12	5	3	4	
Mucin				
(+), *n* = 14	2	1	11	0.014
(−), *n* = 12	4	5	3	
Neutrophil infiltration				
(+), *n* = 9	0	1	8	0.006
(−), *n* = 17	6	5	6	
Necrosis				
(+), *n* = 6	2	0	4	0.794
(−), *n* = 20	4	6	10	

All values are means ± SD.
